# High serum high-density lipoprotein-cholesterol is associated with memory function and gyrification of insular and frontal opercular cortex in an elderly memory-clinic population

**DOI:** 10.1016/j.nicl.2019.101746

**Published:** 2019-03-05

**Authors:** Ryuta Kinno, Yukiko Mori, Satomi Kubota, Shohei Nomoto, Akinori Futamura, Azusa Shiromaru, Takeshi Kuroda, Satoshi Yano, Seiichiro Ishigaki, Hidetomo Murakami, Yasuhiko Baba, Kenjiro Ono

**Affiliations:** a Department of Neurology, Showa University Fujigaoka Hospital, 1-30 Fujigaoka Aoba-ku, Yokohama-Shi, Kanagawa 227-8501, Japan; b Division of Neurology, Department of Medicine, Showa University School of Medicine, 1-5-8 Hatanodai Shinagawa-ku, Tokyo 142-8666, Japan; c Division of Neurology, Department of Internal Medicine, Showa University Northern Yokohama Hospital, 35-1 Chigasaki-chuo Tsuzuki-ku, Yokohama-Shi, Kanagawa 224-8503, Japan

**Keywords:** Elderly, Frontal opercular cortex, Gyrification, High-density lipoprotein cholesterol, Insular cortex, Memory

## Abstract

The issue of whether serum lipid marker values are cognitively and neurologically significant for elderly individuals attending a memory clinic has been controversial. We investigated the associations of serum lipid markers with the memory function and cortical structure in 52 patients aged ≥75 years who had attended our memory clinic based on their subjective memory complaints. None had a history of medication for hyperlipidemia. The Wechsler Memory Scale-Revised (WMS-R) was administered to all patients for the assessment of their memory function. Serum low-density lipoprotein cholesterol, high-density lipoprotein cholesterol (HDLC), and triglyceride (TG) were measured for each patient. Surface-based morphometry (SBM) was performed for the calculation of each patient's cortical thickness and gyrification index based on structural MRI data. Our analyses revealed that the serum HDLC level was positively and significantly correlated with the WMS-R subtests of visual paired associates I/II and logical memory I (p < 0.05). The serum TG level was negatively correlated with the logical memory I subtest. The SBM results showed positive correlations between the serum HDLC level and the gyrification indices of the bilateral insular and frontal opercular cortices, and those two gyrification indices were positively correlated with the logical memory I and visual paired associates I/II. These results suggest that in these elderly patients, a high serum HDLC level was associated with not only preserved memory function but also gyrification of the insular and frontal opercular cortex. We conclude that elderly individuals' serum lipid markers should be carefully assessed in memory clinic settings, because serum HDLC may be a biomarker for memory function and cortical structure.

## Highlights

• HDLC positively correlates with WMS-R scores in an elderly memory clinic population.
• HDLC is associated with gyrification of the bilateral insular and frontal operculum.
• Gyrification of the insular and frontal operculum is associated with WMS-R scores.
• HDLC may be a clinically useful biomarker for an elderly memory-clinic population.

## 1 Introduction

Dementia is characterized by memory loss, cognitive decline, and disability in daily activities. Vascular risk factors such as hypertension, hyperlipidemia, diabetes mellitus, overweight, and smoking are known to enhance the risk of dementia (Blom et al., 2013). Among these factors, the association between hyperlipidemia and dementia remains particularly unclear. Epidemiologic studies examining the association between cholesterol and dementia have reported conflicting results, including an association between adverse hyperlipidemia and an increased dementia risk (Kivipelto et al., 2005; Whitmer et al., 2005), the absence of such an association (Li et al., 2005; Muller et al., 2007b), and inverse associations (Mielke et al., 2005; Reitz et al., 2010). One of the important differences among these studies is the timing of the measurement of serum lipid markers. Significant associations between hyperlipidemia and dementia are described in studies where the subjects' serum lipid levels were measured in midlife (Whitmer et al., 2005). Studies with serum lipid levels measured in later life showed no association (Li et al., 2005) or an inverse association with dementia risk (Mielke et al., 2005). It has been speculated that the association between hyperlipidemia and dementia is affected by several factors, such as apolipoprotein E (APOE) ε4 and the type of dementia (Dufouil et al., 2005; Yasuno et al., 2012). Because dementia typically affects elderly people and is a leading contributor to disability and dependence (Sousa et al., 2009), it is crucial to clarify whether the measurement of elderly individuals' serum lipid markers is cognitively and neurologically useful.

Neuroimaging is one possible method for gaining neurological insights related to dementia. The use of structural magnetic resonance imaging (MRI) has supported clinical diagnoses in memory clinic settings by identifying certain patterns of atrophy and vascular damage (Wattjes, 2011). Several techniques for assessing cerebral morphology such as voxel-based morphometry (VBM) (Ashburner and Friston, 2000) and cortical pattern matching (Thompson et al., 1998) are also available. Surface-based morphometry (SBM) has been used to examine the relationship between cortical thickness and cognitive profiles (Fischl and Dale, 2000; Fjell et al., 2006). As SBM provides more precise measurements (e.g., the actual thickness of the cortex in millimeters), its use makes it possible to analyze the correlation between cognitive abilities and the depth of the cortex across the entire surface of the brain. Several studies have demonstrated that cortical thickness assessed by SBM is significantly correlated with the memory function (Engvig et al., 2010; Palacios et al., 2013; Ystad et al., 2009). SBM can therefore be used to assess the association between serum lipid markers and neurological parameters related to dementia.

SBM can be used to estimate not only cortical thickness but also cortical folding, i.e., gyrification (King et al., 2009). Gyrification exhibits structurally complex patterns (Welker, 1990) that are thought to reflect underlying connectivity (Van Van Essen, 1997). Several methods are used for the assessment of gyrification, and the complexity of gyrification has been shown to be negatively associated with both aging (Kochunov et al., 2005; Liu et al., 2010), and cognitive functions in the elderly (Liu et al., 2011). Changes in gyrification in mild cognitive impairment (MCI) and Alzheimer's disease (AD) have been reported (Im et al., 2008; King et al., 2010), in which the sulci of patients with MCI or AD had less curvature and depth compared to the sulci of healthy subjects. Considering these neurological and cognitive effects, we hypothesized that gyrification is associated with serum lipid markers in elderly people.

We thus conducted the present study to determine whether the measurement of serum lipid markers is cognitively and neurologically useful for an elderly memory clinic population. We retrospectively examined the data of elderly individuals who attend a memory clinic due to their subjective memory complaints. We assessed the neurological and cognitive significance of serum lipid markers by analyzing the elderly patients' cortical structure and memory function. Because the use of a lipid-lowering agent may be associated with a lower risk of dementia in individuals <80 years old (Rockwood et al., 2002), we selectively examined individuals who had no history of medication for hyperlipidemia. Our findings provide new evidence that is applicable in clinical practice for elderly individuals attending a memory clinic.

## 2 Patients and methods

### 2.1 Participants

We retrospectively analyzed the cases of 52 individuals ≥75 years old (82.7 ± 4.86, range 75–93 years); 21 males and 31 females with 12.3 ± 2.66 years of education who had attended the Division of Neurology in the Department of Medicine at the Showa University School of Medicine because of subjective memory complaints. The following four conditions were the inclusion criteria for the study: (1) right-handedness; (2) no history of neurological and neuropsychiatric diseases, including cerebrovascular disease; (3) no medication for hyperlipidemia; and (4) no medical problems related to MRI acquisition. For our evaluations of the significance of the values of serum lipid markers in a memory clinic setting, all of the individuals who met the criteria were examined, regardless of their disease profiles.

The main cause of dementia was AD in 24 patients, MCI in 15, vascular dementia (VaD) in two, diffuse Lewy body disease (DLB) in two, frontotemporal dementia (FTD) in three, and idiopathic normal pressure hydrocephalus (iNPH) in one patient. We also evaluated the cases of five healthy individuals who had attended our memory clinic due to subjective memory complaints and were diagnosed as neurologically normal. Each diagnosis was based on the following diagnostic criteria: the Diagnostic and Statistical Manual of Mental Disorders, 5th Edition (American Psychiatric Association, 2013); the U.S. National Institute on Aging-Alzheimer's Association workgroup for MCI and AD (Albert et al., 2011; McKhann et al., 2011); the Third Report of the DLB Consortium for DLB (Mckeith et al., 2005); the criteria for vascular dementia from the International Society for Vascular Behavioral and Cognitive Disorders (Sachdev et al., 2014); the International Behavioral Variant Frontotemporal Dementia Criteria for FTD (Lamarre et al., 2013; Rascovsky et al., 2011); and frequently used criteria for iNPH (Relkin et al., 2005).

Serum low-density lipoprotein cholesterol (LDLC), high-density lipoprotein cholesterol (HDLC), and triglyceride (TG) were measured for each participant. Each patient's alcohol intake was assessed by self-report, in which there were no patients who drank >20 g/day for men and > 10 g/day for women (43 patients reported no history of drinking, eight reported social drinking, and one male reported <20 g/day). We used the Wechsler Memory Scale-Revised (WMS-R) for the assessment of memory function (Wechsler and Stone, 1987). Approval for this study was obtained from the Institutional Review Board of the Showa University School of Medicine. We provided the participants, their closest family member, or legal guardian the opportunity to opt out of the study.

### 2.2 MRI data acquisition

The structural MRI scans were conducted on a 1 .5T MR scanner (Magnetom Essenza, Siemens, Germany). The high-resolution T1-weighted images of the whole brain (144 sagittal slices, 1.0 × 1.0 × 1.25 mm) were acquired from all of the participants with a gradient echo sequence: repetition time = 1600 msec, echo time = 4.7 msec, flip angle = 15°, field of view = 256 × 256.

### 2.3 SBM analysis

We performed the SBM analysis using the CAT12 Toolbox (http://dbm.neuro.uni-jena.de/cat/) in SPM12 (Wellcome Trust Centre for Neuroimaging, http://www.fil.ion.ucl.ac.uk/spm/) (Friston et al., 1995) implemented on MATLAB R2017b software (MathWorks, Natick, MA, USA). The CAT12 Toolbox contains a processing pipeline for SBM, which includes an established novel algorithm for extracting the cortical surface (Dahnke et al., 2013), which then allows the computation of multiple morphometric parameters (including cortical thickness as well as gyrification index).

For the estimation of white matter distances, we subjected the T1-weighted images to tissue segmentation. Local maxima were then projected to other gray matter voxels by using a neighbor relationship described by the white matter distance (Dahnke et al., 2013). These values equal cortical thickness. This projection-based method also includes partial volume correction, sulcal blurring, and sulcal asymmetries without sulcus reconstruction. Topological correction is performed through an approach based on spherical harmonics. For inter-patient analyses, an algorithm for spherical mapping of the cortical surface is included (Yotter et al., 2011). An adapted volume-based diffeomorphic anatomical registration through the exponentiated lie algebra (DARTEL) algorithm was then applied to the surface for spherical registration (Ashburner, 2007).

In addition to cortical thickness analysis, we extracted the local gyrification index based on the absolute mean curvature (Luders et al., 2006). Central cortical surfaces were created for both hemispheres separately. Finally, all scans were re-sampled and smoothed with a Gaussian kernel of 15 mm full-width at half-maximum (FWHM) for the cortical thickness and 20 mm FWHM for the gyrification index.

### 2.4 Statistical analysis

We used R (ver. 3.5.0) software for the statistical analyses of the patients' and controls' clinical and neuropsychological features. Regarding the SBM analysis, we applied the general linear models to the individual maps and then performed a multiple regression analysis on the individual cortical thickness and gyrification index maps. The serum TG, LDLC, and HDLC levels were then included as covariates in the design matrix of the SBM analysis. Age was included as a nuisance factor in order to correct for the age differences. For the multiple regression analysis, we used threshold-free cluster enhancement (TFCE) (Smith and Nichols, 2009) with 10,000 permutations to identify the brain regions that were significantly associated with serum lipid markers at p < 0.05, after correcting for multiple comparisons across space using family-wise error (FWE) correction. The anatomical locations of the significant clusters were determined with reference to the multi-modal analyses of magnetic resonance images from the Human Connectome Project (HCP) (Glasser et al., 2016).

## 3 Results

### 3.1 The patients' demographics

Table 1 summarizes the demographics of the 52 elderly patients. Eighteen patients (six males, 12 females) showed high serum TG levels (i.e., >149 mg/dl). Five patients (four males, one female) showed low serum HDLC levels (i.e., <40 mg/dl). Thirteen patients (three males, 10 females) showed high serum LDLC levels (i.e., >139 mg/dl). The serum LDLC levels of the female participants were significantly higher than those of the males (p = 0.0014), whereas the serum TG and HDLC levels showed no such differences between the genders. The Spearman partial rank correlation analysis in which gender differences were controlled showed no correlation between any serum lipid markers and age (all, p > 0.05). The duration of education was not correlated with the serum lipid markers (all, p > 0.05). Regarding the WMS-R, the subtest scores were not affected by gender (Table 1). Age and duration of education were not correlated with the subtest scores (Spearman, all, p > 0.05).

**Table 1:** Demographics of the 52 elderly participants.

	Female (n = 31)	male (n = 21)	Statistical value
Age	82.6 ± 0.88	82.9 ± 1.07	Z = 0.21, p = 0.83
Education	12. 8 ± 2.78	11.5 ± 2.32	Z = 1.78, p = 0.076

Serum lipid markers			
TG (50–149 mg /dl)	137.8 ± 74.89	153.0 ± 129.95	Z = 0.18, p = 0.86
HDLC (male: 40–80; female: 40–90 mg /dl)	63.5 ± 12.48	57.4 ± 16.75	Z = 1.43, p = 0.15
LDLC (70–139 mg /dl)	126.2 ± 27.20	99.7 ± 29.04	Z = 3.20, p = 0.0014⁎

WMS-R			
Visual Reproduction I	18.6 ± 8.98	17.5 ± 10.18	Z = 0.48, p = 0.63
Visual Reproduction II	5.6 ± 8.30	5.6 ± 10.08	Z = 0.67, p = 0.50
Logical Memory I	8.5 ± 5.93	7.6 ± 6.51	Z = 0.52, p = 0.61
Logical Memory II	3.0 ± 4.40	2.9 ± 5.3	Z = 0.69, p = 0.49
Verbal Paired Associates I	7.9 ± 6.08	5.8 ± 5.0	Z = 1.29, p = 0.20
Verbal Paired Associates II	3.2 ± 2.88	2.6 ± 2.22	Z = 0.75, p = 0.45
Visual Paired Associates I	4.7 ± 4.76	3.5 ± 4.2	Z = 0.85, p = 0.40
Visual Paired Associates II	1.9 ± 2.12	1.9 ± 2.00	Z = 0.11, p = 0.91
Design memory	4.4 ± 1.89	4.2 ± 1.76	Z = 0.029, p = 0.98
Digit span	10.2 ± 2.70	10.8 ± 4.06	Z = 0.85, p = 0.39
Mental control	3.1 ± 1.53	3.1 ± 1.79	Z = 0.087, p = 0.93
Visual span	12.3 ± 2.45	13.2 ± 4.11	Z = 1.45, p = 0.15

*Z*-values are by Wilcoxon rank sum test.

⁎ p < 0.05.

### 3.2 Correlation analysis between WMS-R and the serum lipid markers

We next examined whether serum lipid markers are associated with memory function. We performed a Spearman partial rank correlation analysis in which the correlation between serum lipid markers and WMS-R subtests was evaluated while controlling for age and gender differences (Table 2). We found that the serum HDLC level was positively correlated with the WMS-R scores on the subtests of visual paired associates I, visual paired associates II, and logical memory I, whereas the serum TG level was negatively correlated with the logical memory I score (all, corrected p < 0.05). In contrast, we found no correlations between the serum LDLC level and the WMS-R subtest scores (all, corrected p > 0.05). These results suggest that both a high level of serum HDLC and a low level of TG were associated with preserved memory function of the elderly memory clinic population.

**Table 2:** Correlation coefficient between serum lipid markers and the WMS-R subtests.

		HDLC	LDLC	TG
	Mean ± SD	61.1 ± 14.52 (mg/dl)	115.5 ± 30.63 (mg/dl)	144.0 ± 99.89 (mg/dl)
Visual Reproduction I	18.2 ± 9.40	0.0036	0.09	0.16
Visual Reproduction II	5.6 ± 8.96	0.25	0.004	−0.18
Logical Memory I	8.1 ± 6.12	0.48⁎	0.18	−0.38⁎
Logical Memory II	2.9 ± 4.74	0.20	0.27	−0.17
Verbal Paired Associates I	7.0 ± 5.71	0.22	0.10	−0.18
Verbal Paired Associates II	3.0 ± 2.63	0.18	0.09	−0.20
Visual Paired Associates I	4.2 ± 4.54	0.32⁎	0.06	−0.24
Visual Paired Associates II	1.9 ± 2.01	0.36⁎	0.20	−0.24
Design Memory	4.3 ± 1.82	0.03	0.04	−0.13
Digit Span	10.4 ± 3.29	−0.07	0.02	0.003
Mental Control	3.1 ± 1.62	0.02	0.08	0.01
Visual Span	12.7 ± 3.22	−0.03	0.18	0.23

Spearman's ρ-values are shown. Age and gender differences were controlled.

⁎ p < 0.05 (Bonferroni correction).

### 3.3 SBM analysis for serum lipid markers

**Fig. 1:**
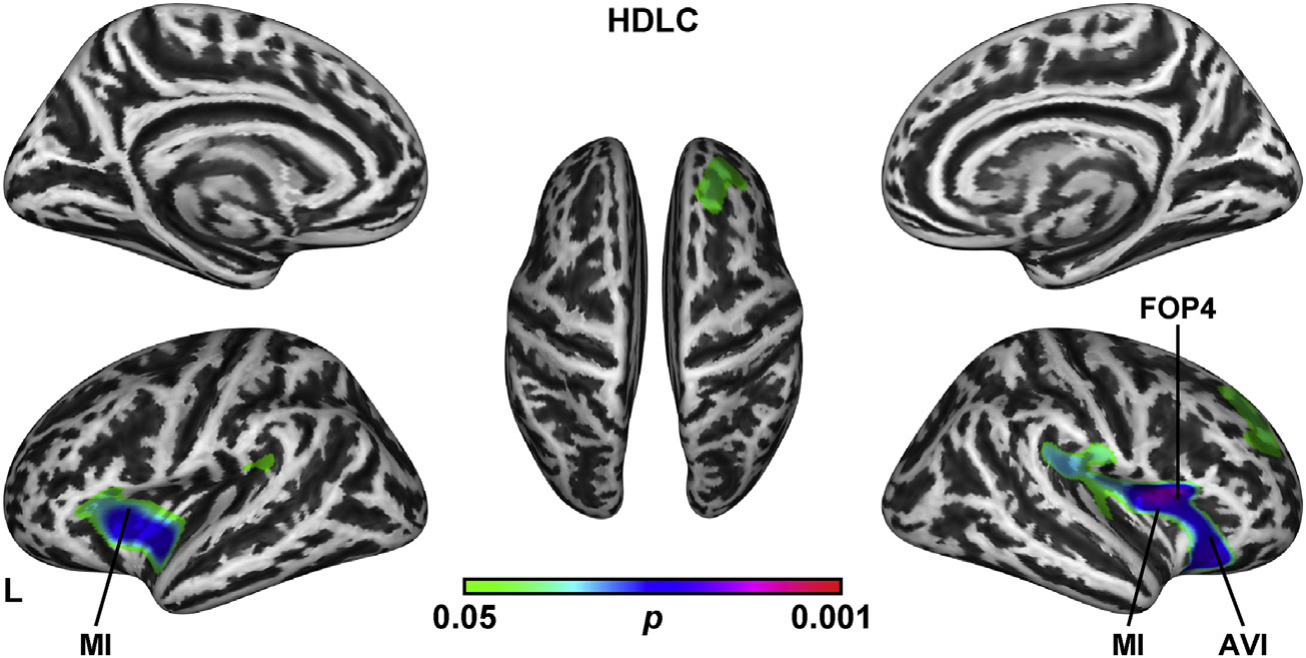
Brain regions with a significant association between the cortical gyrification index and the serum HDLC level. Significant regions are identified by SBM, which was projected onto the left and right lateral surfaces of the standard inflated brain. Medial sections are also shown. The threshold was set at p < 0.05 (TFCE, FWE-corrected). See Table 3 for the details of the regions. Note that no significant correlation was observed for the serum TG level. AVI: anterior ventral insular area, FOP4: frontal opercular area 4, L: left, MI: middle insular area.

We next examined the association of cortical structure with serum HDLC and TG levels, both of which were significantly correlated with memory function (Table 2). The SBM analysis showed that the serum HDLC level was positively associated with the gyrification index in the bilateral insular cortex and frontal opercular cortex (Fig. 1, Table 3). The gyrification indices of the polar frontal cortex, posterior opercular cortex, and premotor cortex also showed weak associations with the serum HDLC level. There were no significant associations of the serum HDLC level with cortical thickness.

**Table 3:** Correlation between the gyrification index and HDLC levels.

P-value	Size	Overlap of atlas region		P-value	Size	Overlap of atlas region	
Left hemisphere				Right hemisphere			
0.02110	754	18%	Middle insular area	0.01580	1678	9%	Area PFcm
		15%	Posterior insular area 2			8%	Frontal opercular area 4
		12%	Area 45			7%	Anterior ventral insular area
		11%	Frontal opercular area 5			7%	Retroinsular cortex
		10%	Posterior insular area 1			6%	Area OP2–3/VS
		9%	Para-Insular area			6%	Area 47 s
		8%	Frontal opercular area 4			5%	Area 44
		6%	Anterior ventral insular area			5%	Frontal opercular area 2
		5%	Area 44			5%	Insular granular complex
		4%	Frontal opercular area 3			5%	Area OP4/PV
0.04920	80	58%	Area PFcm			4%	Area OP1/SII
		24%	Retroinsular cortex			4%	Frontal opercular area 3
		13%	PeriSylvian language area			4%	Frontal opercular area 1
		6%	Area PF complex			3%	Area PF complex
						3%	Frontal opercular area 5
						2%	Rostral area 6
						2%	Area 47 m
						2%	Area 52
						2%	Posterior insular area 2
						2%	Medial belt complex
						2%	Middle insular area
						2%	Area PF opercular
						2%	Anterior agranular insula complex
						1%	PeriSylvian language area
				0.03920	343	32%	Area 9-46d
						29%	Area 8Ad
						18%	Area 9 posterior
						15%	Area 46
						7%	Area 8B lateral

The threshold was set at p < 0.05 (TFCE, FWE-corrected). Each label of the brain regions was based on HCP multi-modal parcellation (Glasser et al., 2016).

Regarding the serum TG level, we found no significant association with cortical thickness or gyrification. These results suggest that the serum HDLC level was not only cognitively but also neurologically associated with preserved memory function of the elderly memory clinic population.

### 3.4 Correlation analysis between the gyrification index and WMS-R scores

We also investigated whether a change in the gyrification index reflects cognitive changes. We performed a Spearman partial rank correlation analysis with which we evaluated the correlation of the WMS-R subtest scores for logical memory I, visual paired associates I, and visual paired associates II with the gyrification index in the regions that showed significant associations with the serum HDLC level (Fig. 1, Table 3). For each region of interest, the mean gyrification indices were extracted based on HCP multi-modal parcellation (Glasser et al., 2016).

**Fig. 2:**
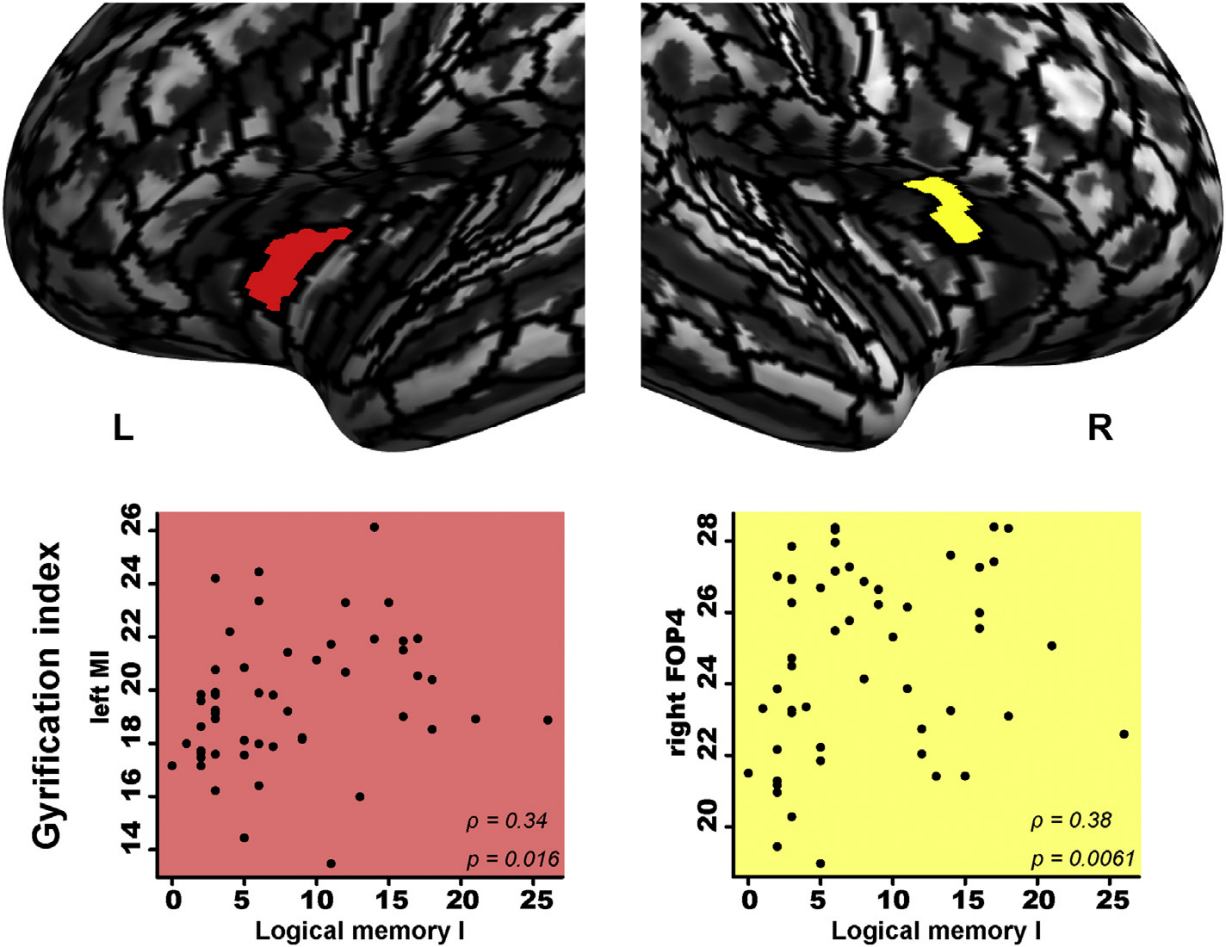
Scatterplots of the association of the gyrification index of the insular and front operculum with the patients' scores on the logical memory I subtest. Spearman partial rank correlation tests showed a significant and positive correlation for each pair (each, p < 0.05). Each label of the brain regions was based on HCP multi-modal parcellation (Glasser et al., 2016). The gyrification index of the left middle insular area and the right frontal opercular area 4 were significantly correlated with the scores on the logical memory I subtest, which are shown in *red* and *yellow*, respectively. FOP4: frontal opercular area 4, L: left, MI: middle insular area, R: right. (For interpretation of the references to color in this figure legend, the reader is referred to the web version of this article.)

**Fig. 3:**
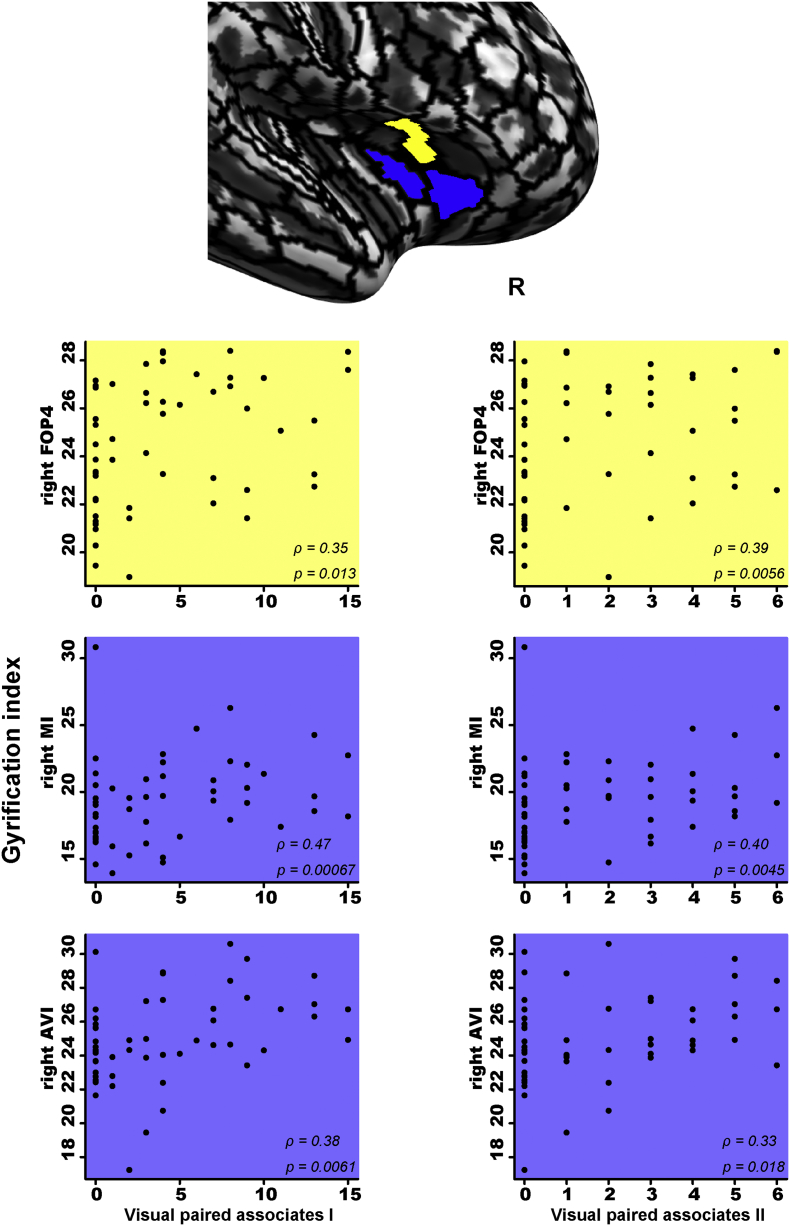
Scatter plots of the association of the gyrification index of the insular and front operculum with the patients' scores on the visual paired associates I and II subtests. The gyrification index of right Frontal Opercular Area 4 was significantly correlated with the score of visual paired associates I and II, which is shown in *yellow*. The gyrification indices of the right middle insular area and right anterior ventral insular area were significantly correlated with the scores on the visual paired associates I/II subtests, which are shown in *purple*. AVI: Anterior ventral insular area; FOP4: Frontal opercular area 4; MI: Middle insular area; R: right. (For interpretation of the references to color in this figure legend, the reader is referred to the web version of this article.)

The score on the logical memory I subtest was significantly and positively correlated with the gyrification index of the right frontal opercular cortex (Fig. 2). The gyrification index of the posterior superior portion of the left short insular gyrus (middle insular area) was also correlated with the logical memory I score. The scores for the visual paired associates I and II subtests were positively correlated with the gyrification indices of the right frontal opercular and right insular cortex (Fig. 3). These results suggest that increased gyrification indices of the bilateral insular cortex and frontal opercular cortex reflect better memory function of elderly individuals.

## 4 Discussion

Our analyses revealed a significant association between two serum lipid markers and the memory function of our patients; i.e., 52 patients aged ≥75 years who had attended our memory clinic based on their subjective memory complaints. We observed that the patients' serum HDLC and TG levels correlated with their scores on three subtests of the WSM-R: visual paired associates I, visual paired associates II, and logical memory I (Table 2). Among the lipid markers, the serum HDLC levels were positively associated with the gyrification of the bilateral insular and frontal opercular cortex (Fig. 1, Table 3). Our findings further showed that the gyrification of the bilateral insular cortex and frontal opercular cortex was clearly associated with the patients' memory function measured by the visual paired associates I, visual paired associates II, and logical memory I subtests (Fig. 2, Fig. 3). These results suggest that the serum HDLC level was associated not only with memory function but also the gyrification of the insular and frontal opercular cortices of an elderly memory clinic population.

The associations between serum lipid levels and cognitive function have been studied by several research groups, and their findings have conflicted. Some studies demonstrated an association between a higher level of serum HDLC and better memory function (Bates et al., 2017; Singh-Manoux et al., 2008; Van Exel et al., 2002). A recent study indicated that persistently high levels of psychological well-being measures predicted a high level of serum HDLC and a low level of serum TG, whereas no association was found between psychological well-being measures and the serum LDLC level (Radler et al., 2018). These findings are consistent with our present results (Table 2).

Regarding the association between the serum LDLC level and cognitive functions, several research groups have reported conflicting results, including both a negative association (Henderson et al., 2003) and a positive association (Leritz et al., 2016). LDLC transports lipids (fats) extracellularly and is associated with an increased risk of cardiovascular disease (e.g., atherosclerosis) (Manninen et al., 1992). Since cerebrovascular and Alzheimer's pathological changes frequently coincide in cases of dementia and may synergistically affect an individual's cognitive decline (Xuereb et al., 2000), it is possible that a high LDLC level is associated with poor cognitive performance. On the other hand, cholesterol is an essential structural component in the cell membranes (Ikonen, 2008), and it is thus theoretically possible that the low LCLC level may be associated with poor cognitive function (Wagstaff et al., 2003). Our present analyses revealed no association between the serum LDLC level and memory function. This difference in results may be due to the characteristics of our patients, who were all ≥75 years old and had no history of hyperlipidemia medication. The serum HDLC level may be especially important for such an elderly memory-clinic population.

Our results demonstrated that the serum HDLC level is associated not only with memory function (Table 2) but also with cortical structure in an elderly memory-clinic population (Fig. 1, Table 3). The evidence of HDLC's involvement in the brain as a whole remains rather limited (Yates et al., 2012). A positive relationship between the serum HDLC level and gray matter volume in the bilateral temporal regions was reported (Ward et al., 2010; Wolf et al., 2004). These studies focused on the gray matter volume, and to our knowledge, no study has investigated the association between gyrification and the serum lipid level. Gyrification is known to be affected by both age and gender (Liu et al., 2010). However, our findings are not likely to be explained by the differences in age and gender, as we controlled our analyses for these differences. We therefore propose that the gyrification change of the insular and frontal opercular cortex is related to the serum HDLC level.

HDLC contains APOE and facilitates the transport of other types of cholesterol from various tissues, including the brain, to the liver. APOE has a major physiological role in the regulation of lipid and lipoprotein homeostasis, and the APOE-ε4 isoform is a well-known risk factor for AD (Raber et al., 2004). Several findings indicate that the association between APOE-ε4 and low HDLC level increases the susceptibility to AD (Hoshino et al., 2002; Raygani et al., 2006). Amyloid β-protein (Aβ), a pathological hallmark of AD, binds to HDLC, maintaining its solubility in cerebrospinal fluid and plasma. This HDLC-Aβ interaction prevents the deposition of Aβ into the brain and can serve as a marker for neurodegenerative disease (Koudinov et al., 2001). APOE-ε4 has been shown to adversely affect this HDLC-Aβ interaction and has been implicated as a risk factor for cerebral amyloid angiopathy, another prominent pathologic hallmark of AD (Mulder and Terwel, 1998). The target of the present study was an elderly memory-clinic population, and therefore, no data of the APOE genotype were available in our present patients, as the use of APOE genotyping is not recommended in routine clinical diagnosis (Farrer et al., 1995). However, considering that the insular cortex is often involved in AD (Bonthius et al., 2005) and the lower gyrification index in AD (Im et al., 2008; King et al., 2010), it should be noted that the APOE genotype may affect the HDLC-Aβ interaction, which would result in the decreased gyrification of insular and frontal opercular cortex.

Gyrification of the insular and frontal opercular cortices was positively correlated with the patients' scores on WMS-R subtests (Fig. 2, Fig. 3). The insular and frontal opercular cortex shows similar recruitment for cognitive function (Duncan and Owen, 2000). Indeed, both regions have been shown to be involved in memory function. The insular cortex was described as involved in recognition memory (Bermudez-Rattoni et al., 2005; Zhao and Wang, 2018). Patients with left insular lesions showed significantly poorer verbal memory as measured by the WMS-R logical memory I subtest, indicating that left insular damage is associated with poorer performance on verbal memory tasks (Manes et al., 1999). The frontal operculum is also known to be involved in several types of memory such as semantic memory and episodic memory (Andreasen et al., 1995; Lepage et al., 2000). These two regions are thought to be connected to each other, as reported regarding cortico-cortical evoked potentials (Enatsu et al., 2016). Indeed, a number of studies have reported the association of these two connected regions with strategic processing during episodic retrieval and working memory (Donaldson et al., 2010; Kahn et al., 2004). A VBM analysis also showed a significant gray matter intensity decrease that covaried with the episodic memory recall performance of patients with familial behavioral variant FTD (Irish et al., 2013), indicating the importance of this connectivity for memory function. Taking the above findings together, we consider that gyrification change of the insular and frontal opercular cortex is related to memory function.

A computer simulation study suggested that thicker cortical sheets lead to less cortical convolutions (Toro and Burnod, 2005). The negative relationship between gyrification and cortical thickness is most likely related to both space constraints and brain functionality. Besides allowing an increase of neuronal numbers within a limited space, another way to increase brain functionality would be to improve the efficiency of cortical communication. Decreasing cortical thickness could shorten the distance of the white matter fibers running between adjacent brain regions, and it could increase the efficiency of cortical communication. Smaller cortico-cortical distances not only aid rapid communication; they also increase the overall efficiency of cortical signaling by reducing the energy expenditure, which may result in better cognitive performance. Indeed, it was reported that greater cortical gyrification is related to better cognitive function, but not to greater cortical thickness (Gautam et al., 2015). Although we observed no significant correlations between critical thickness and memory functions, we suspect that the increased gyrification of the insular and frontal opercular cortex may be reflected by effective cortico-cortical communication, which may contribute to better cognitive performance.

In our elderly memory-clinic population, the WMS-R logical memory and visual paired associate subtest scores were positively correlated with the serum HDLC level (Fig. 2, Fig. 3). Logical memory is known to be used for the assessment of verbal declarative memory (Hori et al., 2013; Muller et al., 2007a; Perry and Hodges, 2000), whereas visual paired associates have been used to assess visual declarative memory (Kessels et al., 2011; Papalambros et al., 2017; Stollery and Christian, 2015). Declarative memory is mediated by the circuitry involving bidirectional connections between the neocortex, the parahippocampal region, and the hippocampus (Eichenbaum, 2000). We recently reported that the scores on the WSM-R logical memory and visual paired associate subtests are positively associated with cortical complexity in the insular and frontal operculum cortex of an elderly memory-clinic population (Kinno et al., 2017). These findings suggest that gyrification change of the insular and frontal opercular cortex is associated with the declarative memory of an elderly memory-clinic population.

This study has some limitations. First, patients with heterogeneous profiles were allowed to participate in the study. In other words, as we aimed to assess the usefulness of the measurement of serum lipid markers for elderly individuals who attended a memory clinic, all participants who met the inclusion criteria (see Methods) were included, regardless of their profiles. However, the disease pattern of our patient series was consistent with that of another study in a memory-clinic setting (Wada-Isoe et al., 2009). Second, as our sample size was relatively small (n = 52), the significance of serum LDLC or TG levels remains unclear. Future studies should clarify the cognitive and neurological significance of these serum lipid markers for specific neurological profiles.

## 5 Conclusion

Our results demonstrate that a high serum level of HDLC was associated with not only preserved memory function but also gyrification of the insular and frontal opercular cortex in an elderly memory-clinic population. This suggests the importance of the assessment of serum lipid markers in clinical practice for the elderly, because their serum HDLC may be a biomarker of both memory function and cortical structure. Further studies are required to establish the significance and the precise effects of an increased serum HDLC level on memory function.

## Declaration of interest

The authors declare no conflict of interest, financial or otherwise, related to this study.
